# Cardiac intimal sarcoma presenting as basilar artery occlusion treated with tenecteplase and thrombectomy: a case report

**DOI:** 10.3389/fcvm.2026.1797980

**Published:** 2026-05-21

**Authors:** Qianqian Wang, Minghui Cheng, Qiang Guo, Ziyou Qi

**Affiliations:** 1 Intensive Care Unit, Department of Neurology, Affiliated Hospital of Jining Medical University, Jining, China; 2 Clinical Medicine Department, Jining Medical University, Jining, China; 3 Department of Emergency Stroke, Affiliated Hospital of Jining Medical University, Jining, China

**Keywords:** basilar artery occlusion, cardiac intimal sarcoma, mechanical thrombectomy, tenecteplase, thrombocytopenia

## Abstract

**Background:**

Cardiac intimal sarcoma is a rare and aggressive malignancy, often presenting with embolic complications such as stroke. Basilar artery occlusion (BAO) is a life-threatening neurological emergency caused by several mechanisms, most commonly embolism (including cardioembolism) and intracranial atherosclerotic disease (with thrombotic occlusion).

**Case presentation:**

A 77-year-old man with a history of coronary heart disease, cerebral infarction, and hypertension presented with acute speech impairment and progressive consciousness disturbance. NIHSS score was 28. The electrocardiogram showed atrial fibrillation. Non-contrast head computed tomography ruled out hemorrhage. Intravenous tenecteplase (22.5 mg) was administered before platelet count results were available, which later revealed thrombocytopenia (54 × 10⁹/L). Emergency mechanical thrombectomy retrieved a 4-cm thrombus from the basilar apex. Histopathology and immunohistochemistry confirmed a diagnosis of intimal sarcoma. Post-procedure, the patient developed multi-organ bleeding and coagulopathy, requiring continuous renal replacement therapy. Despite successful revascularization (NIHSS improved to 10), the patient succumbed to complications of advanced malignancy and hematologic dysfunction.

**Conclusions:**

This case highlights that cardiac intimal sarcoma can present as acute BAO. Histopathological analysis of thrombectomy-retrieved embolic material can establish the diagnosis, obviating the need for invasive cardiac biopsy. Intravenous thrombolysis in the setting of unrecognized thrombocytopenia or malignancy-associated hemostatic dysfunction may carry substantial bleeding risk and warrants careful patient selection, urgent laboratory confirmation when feasible, and multidisciplinary management.

## 1 Introduction

Primary cardiac tumors are rare and complex, with an incidence of approximately 0.02%. The classic clinical triad includes systemic symptoms, cardiac obstruction, and embolic complications (1–3). Among malignant cardiac tumors, sarcomas are the most common histological type, accounting for 75%–95% of primary cardiac tumors (4), and are associated with poor prognosis (5, 6). Cardiac intimal sarcoma is the least reported subtype of primary cardiac sarcoma (7). Approximately 20%–25% of ischemic strokes are cardioembolic, with atrial fibrillation (AF) being the most common cause of cardioembolic stroke (8). Cerebral embolism is the predominant neurological complication of cardiac tumors (1). Tenecteplase is a genetically modified fibrinolytic agent with a longer half-life than alteplase. Tenecteplase at the 0.25 mg/kg dose has symptomatic intracranial hemorrhage rates comparable to those of alteplase and can be administered as a single intravenous bolus. Moreover, randomized trials of intravenous thrombolysis for acute ischemic stroke have reported that tenecteplase and alteplase show no significant differences in 90-day functional outcomes, symptomatic intracranial hemorrhage, death within 90 days, or serious adverse events (9).

## 2 Case

**Figure 1:**
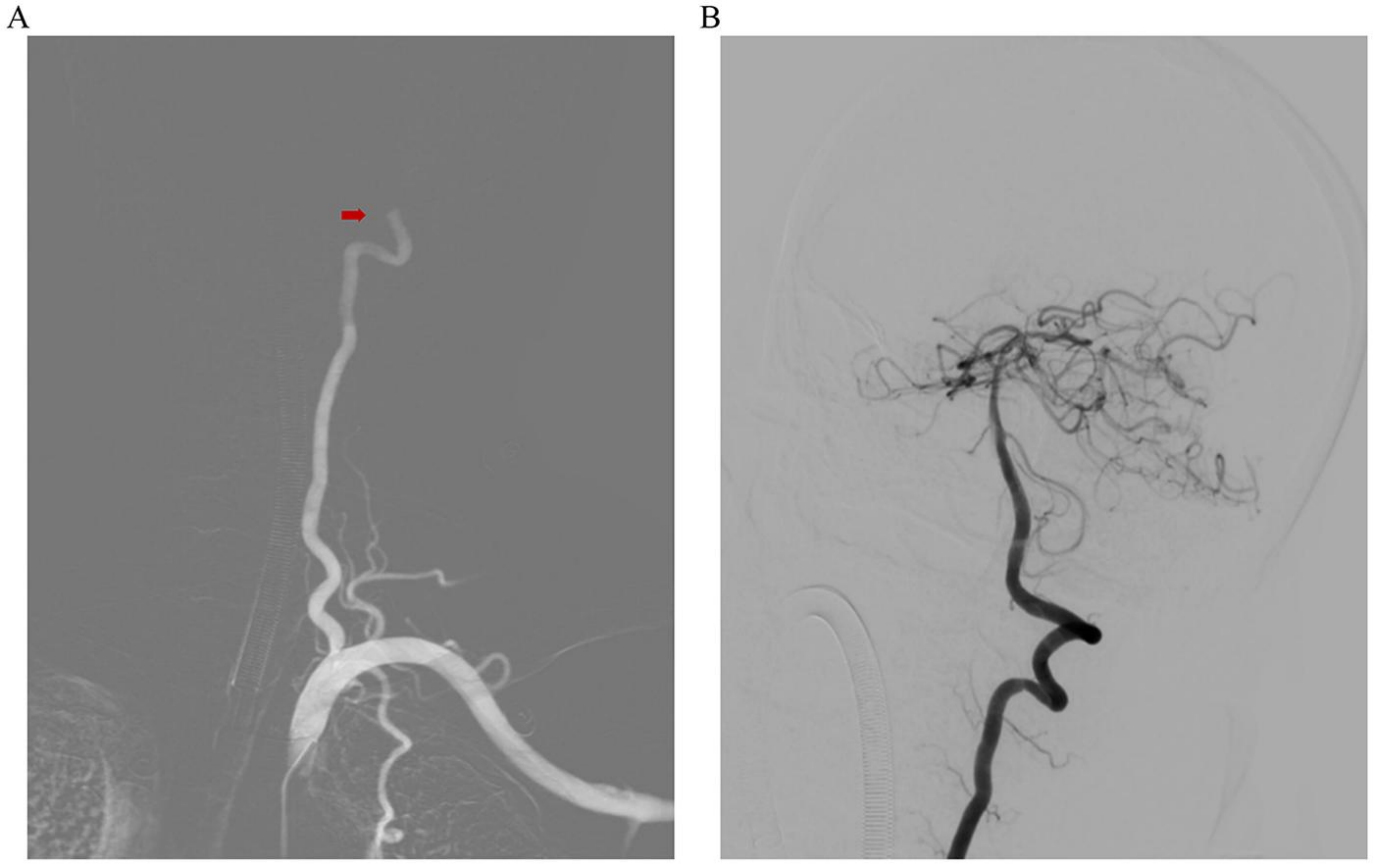
(**A**) Angiography showed patent left vertebral artery (cervical and proximal intracranial segments), with abrupt contrast cutoff at distal V4 (red arrow). (**B**) Post-procedure angiography showed improved flow in both posterior cerebral arteries.

**Figure 2:**
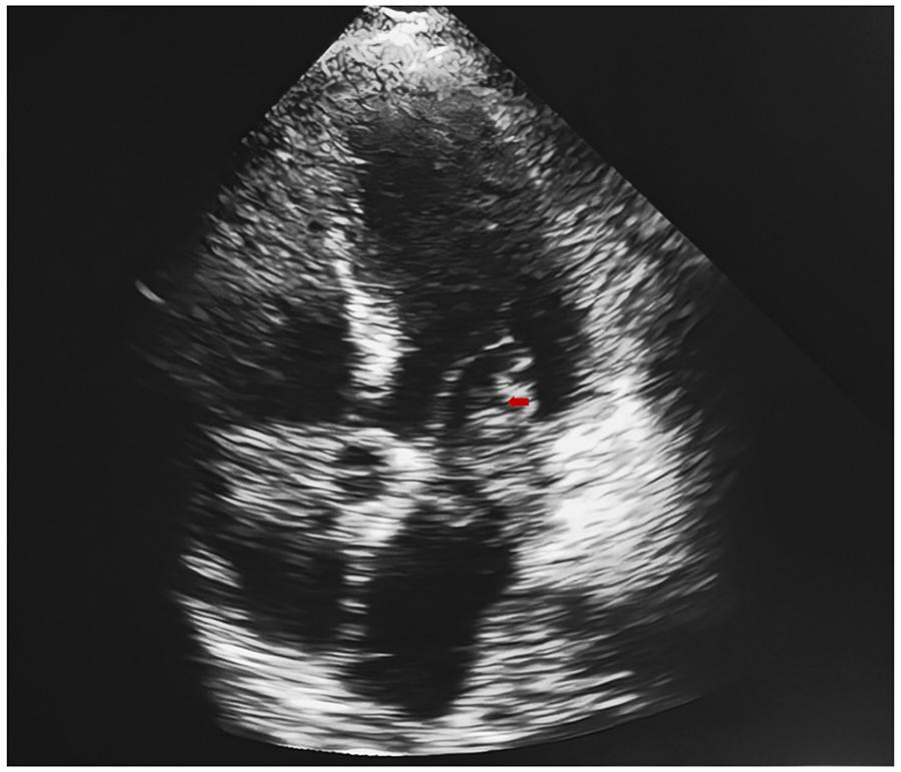
Transthoracic echocardiography shows an echogenic lesion in the left atrium, as indicated by the red arrow.

At 40 min after symptom onset, a 77-year-old man with a history of coronary heart disease, cerebral infarction, and hypertension presented to the neurology emergency department with slurred speech. The patient's body temperature was 36 °C, pulse rate 125 beats per minute, respiratory rate 20 breaths per minute, and blood pressure 168/102 mmHg. The physical examination revealed impairment of consciousness, left limb muscle strength grade 3, right upper limb muscle strength grade 0, right lower limb muscle strength grade 1, decreased muscle tone in all limbs, and poor response to stimulation. Heart rhythm was irregular. The NIHSS score was 28 (consciousness 6, speech 5, motor 16, ataxia 1). The electrocardiogram showed atrial fibrillation (AF). Non-contrast head computed tomography ruled out intracranial hemorrhage, and random blood glucose excluded hypoglycemia. Before the complete blood count results were available, given the disabling deficit and imaging findings, intravenous tenecteplase (22.5 mg) was administered. Shortly thereafter, the platelet count was reported as 54 × 10⁹/L, indicating thrombocytopenia with increased bleeding risk. A white blood cell count of 12.61 × 10⁹/L, with neutrophils 6.18 × 10⁹/L and lymphocytes 5.30 × 10⁹/L were also noted., and a C-reactive protein level of 1.9 mg/L. Arterial blood gas analysis showed pH 7.35, pCO₂ 45 mmHg, and pO₂ 64 mmHg. Lactate, coagulation profile, and cardiac enzymes including troponin I, myoglobin, and CK-MB were all within normal ranges. The patient was transferred immediately to the angiography suite under general anesthesia for emergency endovascular therapy. During the procedure, angiography revealed occlusion at the top of the basilar artery, and a large, elongated thrombus (length 4 cm, diameter 0.1–0.3 cm, medium consistency) was aspirated. Post-procedure angiography showed improved flow in both posterior cerebral arteries (Figure 1), and the NIHSS decreased to 10. On the evening of the same day, the patient developed oronasal and gastrointestinal bleeding. Laboratory tests showed severe coagulopathy: platelets 47 × 10⁹/L, prothrombin time 21.5 s, activated partial thromboplastin time (APTT) 82.7 s, thrombin time 27.5 s, and fibrinogen 0.65 g/L. On the morning of post-procedure day 2, follow-up tests revealed an increasing white blood cell count (17.84 × 10⁹/L), markedly elevated D-dimer (12.52 μg/mL), APTT 51.3 s, fibrinogen 0.60 g/L, and troponin I 5,519 ng/L. Transthoracic echocardiography performed at this time showed a left ventricular ejection fraction of approximately 51%, regional wall-motion abnormalities in the inferior and posterior walls, and an echogenic lesion in the left atrium (Figure 2). Within the next 24 h, hemoglobin fell to 80 g/L, platelets to 24 × 10⁹/L, fibrinogen to 0.60 g/L, D-dimer increased to 13.31 μg/mL, and troponin I rose to >50,000 ng/L, with B-type natriuretic peptide 1,162.30 pg/mL. Further laboratory tests demonstrated normal ionized calcium (1.13 mmol/L), liver function, renal function, ammonia, electrolytes, and serum levels of vitamin B12, folate, and ferritin. The patient was blood type A, Rh(D) positive, and the direct antiglobulin test was negative. On post-procedure day 4, the patient developed rapid AF with hypotension, oliguria, and persistently elevated serum creatinine, necessitating continuous renal replacement therapy (CRRT). After 38 days of intensive care, the patient died. The final diagnosis was basilar artery occlusion secondary to cardiac intimal sarcoma, confirmed by histopathological and immunohistochemical analysis of the embolic material retrieved during mechanical thrombectomy.

### 2.1 Pathology Report

**Specimen**: embolic material retrieved from the basilar apex during mechanical thrombectomy.

**Microscopy**: Hematoxylin and eosin staining of the basilar artery apex showed atypical spindle cells consistent with intimal sarcoma.

**Immunohistochemistry**: CK (scattered +), CDK4 (+), CD34 (-), MDM2 (±), desmin (-), SMA (-), GFAP (-), vimentin (+), Ki-67 (+, hotspot 50%–60%).

**Figure 3:**
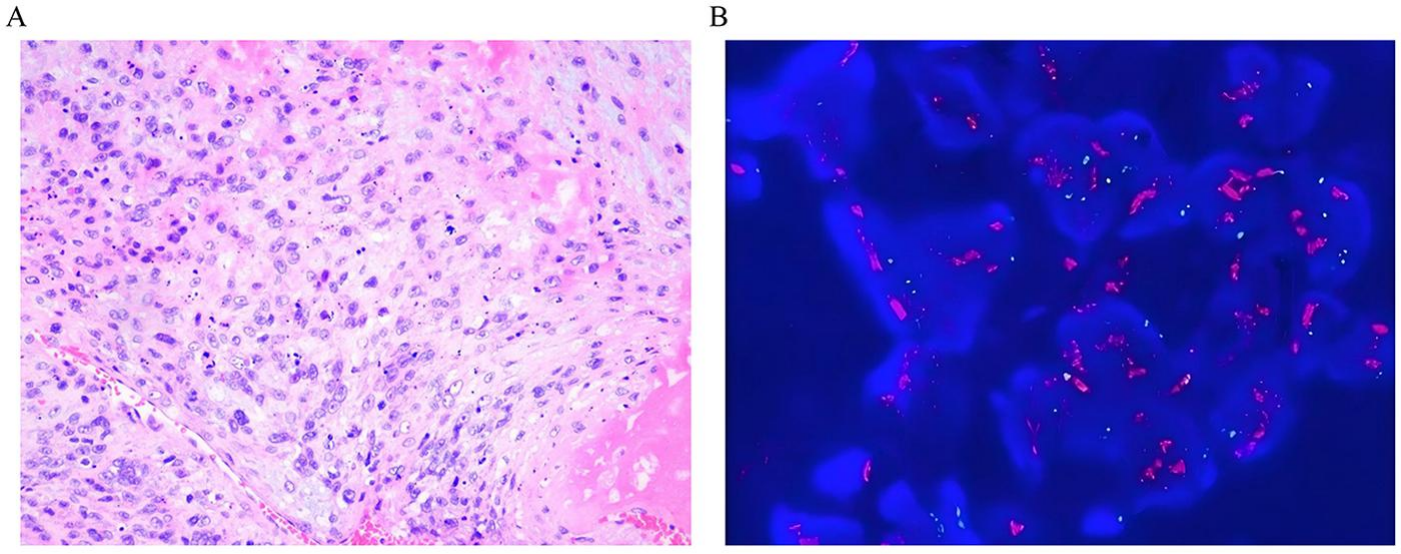
Fluorescence *in situ* hybridization (FISH) shows MDM2 gene amplificatioHistopathological examination of the thrombus extracted via mechanical thrombectomy. **(A)** Hematoxylin and eosin staining reveals atypical spindle cells consistent with sarcoma. **(B)** Fluorescence *in situ* hybridization (FISH) shows MDM2 gene amplification, with the red signal representing the MDM2 probe and blue indicating DAPI-stained cell nuclei.(original magnification   ×  200).

**Ancillary studies**: Fluorescence *in situ* hybridization (FISH) showed an amplified MDM2 gene, which supported the diagnosis of intimal sarcoma (Figure 3).

## 3 Discussion

This case illustrates the intersection of cardiac disease and posterior circulation large-vessel occlusion (2). Neurological complications of cardiac tumors, particularly cerebral embolism, are well documented (1). Cardiac intimal sarcoma is a rare, aggressive subtype of primary cardiac sarcoma and is associated with poor outcomes (4–7).

The patient's wife and his son, a rehabilitation physician, were his family members. Treatment decisions were made with the son's active participation. His son was initially optimistic but was not prepared for the rapid clinical deterioration. It took the family 10–12 days to gradually come to terms with the poor prognosis after the confirmed pathological diagnosis. Families in extremely uncertain situations rely on frequent cues, such as clinical updates and patient responses, to navigate difficult decisions, which is consistent with findings from a qualitative study on proxy decision-making in malignant brain tumors (10). This case highlights the importance of acknowledging the family's evolving understanding and providing ongoing support when clinical circumstances change.

In our patient, a distal BAO produced a posterior circulation syndrome. A thorough diagnostic workup was conducted. The lack of notable atherosclerotic stenosis on DSA refuted large-artery atherosclerosis as the cause of stroke. Infectious endocarditis and vasculitis were ruled out by a comprehensive infectious and immunological workup that included negative blood cultures, normal C-reactive protein levels, and negative autoimmune serologies. A bone marrow examination ruled out primary hematologic disorders as a cause of the thrombocytopenia, suggesting processes associated with malignancy and/or consumption. Several case reports have documented severe thrombocytopenia in association with primary cardiac sarcomas. For example, a left atrial rhabdomyosarcoma (11), a primary cardiac synovial sarcoma (12), and an intimal sarcoma of the inferior vena cava with right atrial extension (13) have all been reported to cause marked thrombocytopenia, with platelet counts rapidly normalizing after tumor resection. Immune-mediated destruction (14) and mechanical shear stress from intracardiac tumor flow disturbances (12–14) have been proposed as potential mechanisms. In our patient, although the platelet count did not recover due to persistent malignancy and consumptive coagulopathy, the initial thrombocytopenia (54 × 10⁹/L) and subsequent rapid decline post-procedure may also be partially attributable to the cardiac intimal sarcoma itself. Together, these findings reinforced the diagnosis of a cardioembolic source, which was determined to be cardiac intimal sarcoma. Thrombolysis with tenecteplase followed by mechanical thrombectomy achieved rapid reperfusion and early neurological improvement (NIHSS 28 → 10). However, the clinical course included multi-organ hemorrhage, severe and rapidly progressing coagulopathy (with APTT rising to 82.7 s and fibrinogen dropping to 0.60 g/L within hours of the procedure), rapid AF with hypotension, elevated troponin, and renal dysfunction necessitating CRRT. The marked increasing in troponin I (>50,000 ng/L), BNP, and D-dimer after surgery probably indicates a combination of stroke-heart syndrome, acute coronary syndrome, and potential tumor embolization or myocardial infiltration, all of which can occur during aggressive malignancies, such as intimal sarcoma. This complexity aligns with the challenging clinical context of malignant cardiac tumors and their management (2). AF contributes to cardioembolic events (8). Pericardial involvement and hemorrhagic complications have been reported in cardiac sarcomas and can worsen cardiocirculatory status. Even successful revascularization may not be able to reverse the underlying disease trajectory in a life-threatening posterior circulation stroke caused by an aggressive and advanced cancer, as highlighted by the case results.

Revascularization can salvage ischemic brain tissue in large-vessel occlusion. Histopathological analysis of the retrieved embolic material has high diagnostic value, confirming intimal sarcoma and potentially reducing reliance on cardiac biopsy. In this case, FISH detection of MDM2 gene amplification in the embolus—a molecular hallmark of intimal sarcoma-confirmed the diagnosis. Cardiac magnetic resonance imaging may enhance the detection of the underlying mass and guide diagnostic and treatment planning (2). However, its viability might be restricted in critically ill patients, such as in this case. Oncologic management involves surgical resection and adjuvant therapy (3, 6). In addition, complex resections are feasible in selected cases (5). Molecular studies have characterized cardiac sarcomas and support classification frameworks that may inform clinical management (4).

Cardiac intimal sarcoma can present as a life-threatening cerebral embolism. Even after successful reperfusion, severe hematologic impairment and multi-organ dysfunction may affect outcomes. Optimal management is multidisciplinary, integrating neurology, cardiology, hematology, intensive care, and pathology, with attention to early tumor detection, reperfusion, and coordinated oncology planning (1–3, 9, 15, 16). Mechanical thrombectomy in patients with thrombocytopenia, as in this case, requires careful consideration due to potential bleeding risks, which has been reviewed in recent literature (17).

## 4 Conclusion

This case underscores that cardiac intimal sarcoma, although rare, can present as life-threatening BAO. Detecting the underlying cause requires a thorough diagnostic evaluation that includes DSA to rule out atherosclerosis, infectious and immunological tests to rule out endocarditis, and bone marrow examination to assess thrombocytopenia. The diagnosis was established by histopathological and immunohistochemical analysis of thrombectomy-retrieved embolic material with confirmatory MDM2 FISH, rather than by cardiac biopsy, highlighting a critical diagnostic pathway in neurovascular emergencies involving previously undiagnosed malignancy. While revascularization with tenecteplase and thrombectomy achieved neurological improvement, the subsequent severe and refractory coagulopathy illustrates the major bleeding risk of systemic thrombolysis in patients with unrecognized thrombocytopenia or malignancy-associated hemostatic dysfunction. A practical constraint in such complicated instances is highlighted by the patient's instability, which makes advanced cardiac imaging impossible. The family's perspective was included to emphasize empathetic communication and shared decision-making in the context of catastrophic illness. The outcomes and risks associated with mechanical thrombectomy in the setting of thrombocytopenia warrant specific attention. Ultimately, the management of such complex cases requires a high index of suspicion for cardioembolic sources, careful weighing of reperfusion risks, and multidisciplinary collaboration among neurology, cardiology, hematology, and oncology teams to guide both acute intervention and subsequent oncologic care.

## Data Availability

The original contributions presented in the study are included in the article/Supplementary Material, further inquiries can be directed to the corresponding author.
